# Changes in behaviour drive inter-annual variability in the at-sea distribution of northern gannets

**DOI:** 10.1007/s00227-016-2922-y

**Published:** 2016-06-18

**Authors:** V. Warwick-Evans, P. W. Atkinson‎, J. P. Y. Arnould, R. Gauvain, L. Soanes, L. A. Robinson, J. A. Green

**Affiliations:** School of Environmental Sciences, University of Liverpool, Liverpool, L69 3GP UK; British Trust for Ornithology, The Nunnery, Thetford, Norfolk, IP24 2PU UK; School of Life and Environmental Sciences (Burwood Campus), Deakin University, Geelong, VIC Australia; Alderney Wildlife Trust, St Annes, GY9 3TA Alderney, Channel Islands; Department of Life Sciences, University of Roehampton, Whitelands Campus, London, SW15 5PU UK

## Abstract

The at-sea distribution of seabirds primarily depends on the distance from their breeding colony, and the abundance, distribution and predictability of their prey, which are subject to strong spatial and temporal variation. Many seabirds have developed flexible foraging strategies to deal with this variation, such as increasing their foraging effort or switching to more predictable, less energy dense, prey, in poor conditions. These responses may vary both within and between individuals, and understanding this variability is vital to predict the population-level impacts of spatially explicit environmental disturbances, such as offshore windfarms. We conducted a multi-year tracking study in order to investigate the inter-annual variation in the foraging behaviour and location of a population of northern gannets breeding on Alderney in the English Channel. To do so, we investigated the link between individual-level behaviour and population-level behaviour. We found that a sample of gannets tracked in 2015 had longer trip durations, travelled further from the colony and had larger core foraging areas and home range areas than gannets tracked in previous years. This inter-annual variation may be associated with oceanographic conditions indexed by the North Atlantic Oscillation (NAO). Our findings suggest that this inter-annual variation was driven by individuals visiting larger areas in all of their trips rather than individuals diversifying to visit more, distinct areas. These findings suggest that, for gannets at least, if prey becomes less abundant or more widely distributed, more individuals may be required to forage further from the colony, thus increasing their likelihood of encountering pressures from spatially explicit anthropogenic disturbances.

## Introduction

It is widely accepted that seabirds have developed flexible foraging strategies as a mechanism with which to respond to seasonal and/or annual variation in the abundance and distribution of prey (Weimerskirch et al. 2005). For example, in response to poor prey availability, seabirds may exploit more predictable prey types, lower in energetic value (Wanless et al. 2005), or they may increase foraging effort (Monaghan et al. 1994). They may do this by varying their time budget while at sea (Ronconi and Burger 2008), or by increasing the duration or range of foraging trips (Garthe et al. 2011; Monaghan et al. 1994; Uttley et al. 1994). However, this variability in foraging behaviour can have consequences for reproductive success (Becker et al. 2007). This is because seabirds are central place foragers during the breeding season, constrained to return to the colony regularly throughout the incubation and chick-rearing period. Thus, increased foraging trip duration may result in both parents undertaking simultaneous foraging trips, leaving eggs or chicks unattended at the nest and subject to attacks by predators or conspecifics (Lewis et al. 2004). Therefore, energy limitation, predation or competition can have implications on reproductive success. Ultimately, as long-lived animals, seabirds will prioritise their own survival over that of their offspring and abandon breeding attempts when prey availability is very low (Ponchon et al. 2014).

Variation in oceanic conditions may influence the spatial or temporal availability of prey (Burke and Montevecchi 2009; Chavez et al. 2003). An example of this is the North Atlantic Oscillation, a climatic event where fluctuations in atmospheric pressure at sea level result in warmer, wetter and windier climates (Hurrell 1995), with warmer sea temperatures in years with a high NAO index (Sims et al. 2001). Years of high NAO have been associated with lower overwintering survival (Votier et al. 2005) and breeding performance of seabirds (Paiva et al. 2013a, b; Thompson and Ollason 2001). While research efforts have focussed on linking variation in oceanographic conditions to productivity at the population level, the role of intra- and inter-individual variation in behaviour has received little attention (Wakefield et al. 2015). Indeed, in most cases variation amongst individuals in the population has been overlooked, under the classical assumption that individuals in a population behave in similar ways. Yet variation in foraging behaviour can occur both within and between individuals (e.g. Barlow and Croxall 2002; Kato et al. 2000; Woo et al. 2008). However, few studies have looked at how intra- and inter-individual variation differs between years, and what the consequences of this may be at a population level.

**Fig. 1:**
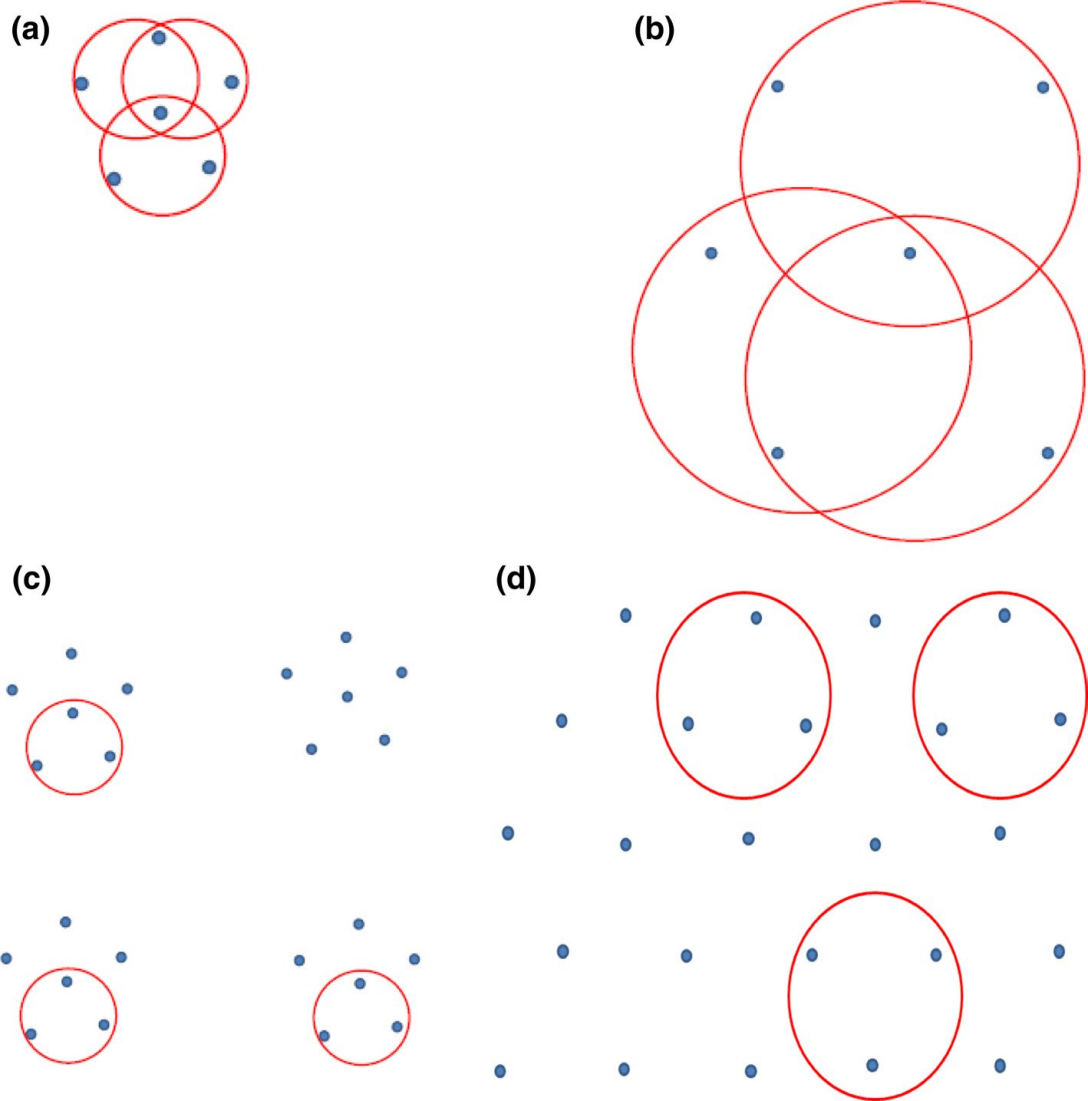
Four hypothetical scenarios to describe the distribution of prey (*blue dots*) and the foraging location of seabirds (*red circles*); **a** low resource + high patchiness = small foraging area and high inter-individual overlap, **b** low resource + low patchiness = large foraging area and high inter-individual overlap, **c** high resource + high patchiness = small foraging area and small inter-individual overlap, **d** high resource and low patchiness = large foraging area and small inter-individual overlap

Inter-annual variation in both the abundance and distribution of prey might lead to variation in inter-individual variability in the size, location and overlap of foraging areas (Fig. 1). Low inter-individual variation in trip duration or foraging area may occur because prey is concentrated in particular areas, attracting all individuals in a population. Alternatively, this may be because prey is sparsely distributed, and all individuals in the population have large searching areas, i.e. all individuals are going everywhere. Alternatively, high inter-individual variation suggests that prey is abundant in their distribution, either patchy or dispersed (Fig. 1). Additionally, intra-individual consistency in foraging locations of seabirds has been observed at various temporal scales across months and years in some individuals, yet others show high intra-individual variability (Ceia and Ramos 2015; Wakefield et al. 2015). These diverging strategies suggest that some individuals in a population may have greater specialisation with regards to diet and habitat use than others (Bearhop et al. 2006). This inter-individual variation is essential to consider when tracking studies are used to identify important areas for conservation, because often only a small proportion of the population is tracked, and few studies take into account how well the sampled individuals represent the foraging locations of the entire population (Soanes et al. 2013a). If the foraging locations of the tracked individuals do not represent those of the entire population, then important at-sea locations may be overlooked, which may be crucial when tracking data are used to identify important areas for marine spatial planning (Soanes et al. 2013a). However, by using what we know about the size and location of the foraging areas of tracked birds, it is possible to incorporate this limitation and predict the size of foraging areas used by the entire population (Soanes et al. 2013a).

Consistency in foraging locations as a result of individual dietary and habitat specialisation has been observed in northern gannets *Morus bassanus* (Patrick et al. 2014; Wakefield et al. 2015). This challenges their traditional classification as generalist predators that feed on a variety of pelagic fish and fisheries discards (Nelson 1978; Votier et al. 2010). Additionally, northern gannets, and congeneric populations, show inter-annual variation in foraging behaviour and reproductive success as a result of sea temperature, primary productivity and the type and abundance of prey (Angel et al. 2015; Garthe et al. 2011; Montevecchi 2007). However, most studies overlook the link between this individual consistency and inter-annual variation. This is important because while northern gannet populations are increasing at an average of 3 % per year across the UK and Ireland (Wanless et al. 2006), they have high conservation importance due to their restricted ranges, with 75 % of the world’s population breeding in Europe (Gremillet et al. 2006). Consequently, there is concern that populations may be impacted by anthropogenic pressures such as over-fishing of prey stocks (Gremillet et al. 2015), changes in the bycatch policy (Votier et al. 2013) or the installation of windfarms (Furness et al. 2013). To understand how gannets are going to be affected by these pressures, a better understanding of inter-annual variation in foraging behaviour at both the individual and the population level is required. For example, if in years of low prey availability all individuals in the population visit larger areas, then all individuals may have an increased risk of interacting with these pressures. Conversely, if inter-annual variation in foraging behaviour is driven by individual birds visiting different areas, then spatial pressures may have differential effects on individuals in the population.

Here we use 4 years of tracking data to investigate the inter-annual variation in the foraging behaviour and space use by a population of northern gannets breeding on Alderney, Channel Islands. Alderney’s population may be particularly vulnerable due to its position near the southern limit of the species range (Brown et al. 1996), the overlap in home range with offshore developments (Soanes et al. 2013b) and the limitation in extending its range due to competition from conspecifics in nearby colonies (Wakefield et al. 2013). We investigate the link between individual-level and population-level behaviour and explore NAO as a potential driver of this variability. Specifically, we determined whether in years where the population has a larger foraging area, if this is driven by individual birds diversifying to visit more different, distinct patches of high prey availability (e.g. Fig. 1d), or by each bird increasing its own foraging area to overlap with the foraging area of the entire population, suggestive of low prey availability (e.g. Fig. 1b).

## Materials and methods

### Data collection

Fieldwork was conducted at the breeding colony of northern gannets on Les Etacs, Alderney (49°42′N, 2°14′W), during the early chick-rearing period in early June of 2011 and 2013–2015. All procedures were licensed by the States of Alderney. Birds with chicks approximately 2–4 weeks old were captured at their nest using a noose pole. GPS data recorders, logging positions every 2 min (IgotU GT 120 (2011), IgotU GT-600 (2013–2015), Mobile Action Technology) packaged in plastic heatshrink, were attached to the base of the tail using Tesa Extra Power tape. The devices weighed 22 g or 33 g, ~1 % of the body mass of an average gannet (3.3 kg, Wanless and Okill 1994). Loggers were removed 2–3 weeks later and birds not recaptured would have lost their devices within approximately 1 month (pers obs). Devices of 1 % body mass have previously been shown to have no effect on foraging duration, breeding success or body condition in northern gannets (Hamer et al. 2000).

Breeding success was monitored at the colony in 2013–2015 as per the UK seabird programme monitoring methods handbook (Walsh et al. 1995). At the start of the chick-hatching period, five plots were designated, each containing 50 Apparently Occupied Sites (AOSs), and the number and age of the chicks were recorded every 7–10 days throughout the breeding season. The number of chicks which fledged in each plot were divided by 50 and averaged across the five plots in order to obtain a value of chicks fledged per pair for the colony. Due to the inaccessibility of the colony, these productivity counts were conducted via a telescope from the main island of Alderney; thus, only nests on the edge of the colony could be observed, probably resulting in a biased sample of newer, less successful breeders (Nelson 2002). Consequently, estimates of fledging success obtained in the present study may not be comparable to those obtained elsewhere. However, this potential bias should remain consistent between years, allowing for inter-annual comparisons on a relative basis.

### Data processing and analysis

GPS positions were interpolated to every 10 s using the *adehabitatLT* package (Calenge 2006) in R (R Core Team 2013) to account for missing data associated with diving behaviour or occasional missed GPS locations. The colony was defined as Les Etacs rocks (49.705 N, 2.239 W) with a 30 m surrounding buffer, based on personal observations of gannet behaviour, and for each bird, each trip was defined as all points between leaving and returning to this area. Trip characteristics including: duration (hours); trip length (total distance, km); maximum distance from the colony (km); and directness (trip length/maximum distance from the colony) were calculated for each trip for each bird. Directness is a measure of deviation from a straight line, with a value of 2 representing direct movement between the colony and furthest point, and anything above this representing a less direct track. A frequency histogram of trip duration showed a clear bimodal distribution. One mode represented trips up to 40 min in duration, whereas the second mode represented trips lasting many hours. Foraging trips were, therefore, defined as any trip over 40 min in duration to discount birds loafing adjacent to the colony, or short periods of flight following disturbance at the colony.

**Fig. 2:**
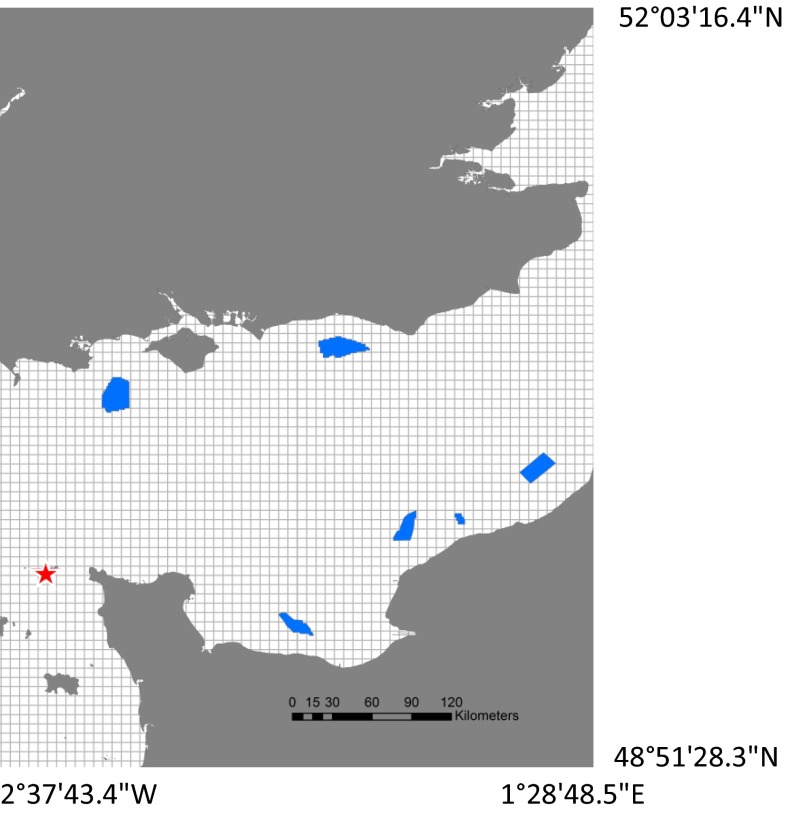
Windfarms proposed for development in the English Channel (*blue*), and the colony of northern gannets breeding on Alderney (*red star*), overlaid with the 5 km × 5 km grid for which time in area was calculated

General linear mixed effects models were used in package *nlme* (Pinheiro et al. 2016) to identify inter-annual variation in trip characteristics. Year was the fixed effect and individuals were included as random effects to account for pseudo-replication. *Post hoc* Tukey tests were conducted in package *multcomp* (Hothorn et al. 2008) to identify between which years differences lay, and least squared means were calculated using package *lsmeans* (Lenth 2016) to calculate annual mean values of all trip characteristics. Warwick-Evans et al. (2015) showed that individual gannets increase time allocation in spatial locations where they forage more frequently, and that 5 × 5 km is the most appropriate scale at which to capture this behaviour. Thus, the R package *Trip* (Sumner 2011) was used to calculate the proportion of time spent (s) in each 5 × 5 km cell of a pre-defined grid around the colony for each bird for each year (Fig. 2). These proportions were then averaged across all birds for each year to define the most important foraging areas for the population. The cells used were ranked in order of time spent and the top 95 % were defined as the home range area (HRA) and the top 50 % the core foraging area (CFA). The CFA and HRA for each year were plotted in ArcGIS (ArcGIS version 10.2), and the size of these areas was calculated. Additionally, the cells in which individual birds spent the top 50 and 95 % of their time were calculated in order to measure inter-individual variation in CFA and HRA. Again, time spent in each grid cell can be used as a proxy for foraging behaviour, because individuals of this species spend more time in areas with increased foraging activity (Warwick-Evans et al. 2015). To quantify the interactions between northern gannets breeding on Alderney, and windfarms proposed for development in the English Channel, the number of foraging trips which overlapped with proposed development sites (downloaded from 4cOffshore 2015) in each year was calculated using ArcGIS (Fig. 2).

In order to calculate how well the individuals that we tracked represented the HRA and CFA of the entire population in a specific year, we followed the methodology devised by Soanes et al. (2013a). For each year independently, the HRA and CFA were calculated initially for one individual and subsequently for an increasing number of individuals. The individuals included in each calculation were sampled at random from all of the tracked birds, until the total number of gannets tracked that year had been sampled. These data were then bootstrapped 10,000 times using R package *boot* (Canty and Ripley 2014) to determine the mean values of CFA and HRA. These data were then fitted to the Michaelis–Menten model as per Soanes et al. (2013a).

$${\text{Michaelis}} {-} {\text{Menten:}}\,y = a \times x/(b \, + \, x)$$

**Fig. 3:**
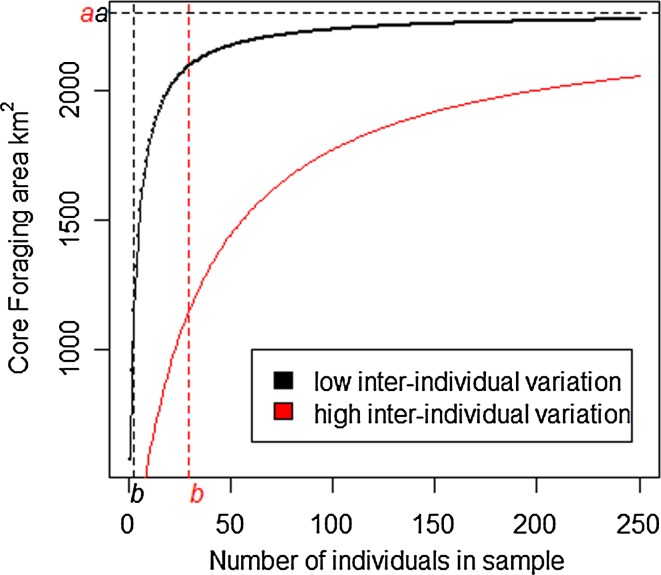
A hypothetical relationship between the number of individuals sampled and the size of the core foraging area for seabirds showing high and low inter-individual variation in core foraging area locations

This model uses information about the size of the CFA and HRA of the tracked birds to predict the size of these areas for an increasing sample size, and ultimately for the entire population. This allows us to extract the asymptotic value of the *y* axis (*a*) i.e. the size of the CFA/HRA predicted for the entire population, and the value at which half of the maximum response is attained (*b*) i.e. the number of individuals necessary to sample in order to represent half of the CFA/HRA for the entire population (Fig. 3). Thus, the value of *b* can be used to describe inter-individual variation in the location of CFAs and HRAs. Values of *a* and *b* were then used to extrapolate the CFA and HRA for the entire population of approximately 10,000 birds breeding on Alderney, for that specific year. We then calculated the proportion of the population-level CFA and HRA that was represented by our sample of gannets for each year independently.

Additionally, this approach can inform us of the number of trips necessary to sample from an individual in order to represent half of its individual CFA or HRA (Soanes et al. 2013a). Thus, at the individual level, *b* can be used to describe intra-individual variation, or consistency, in the location of CFA and HRA. For example, if the entire CFA or HRA of an individual could be determined from just one trip, then the value of *b* would be low, intra-individual variation in terms of the location of CFA or HRA would be low, and consistency would be high. Thus, this approach was used to determine how well the trips we sampled from each individual represented the entire foraging area for that individual, and also how consistent each individual was between trips. In 2011, only four individuals recorded three or more trips; thus, the Michaelis–Menten equation could only be fitted for these four individuals, and conclusions about consistency within individuals in 2011 should be interpreted cautiously.

In order to evaluate the overlap in space use between sampled individuals, the number of birds that used each 5 × 5 km grid cell in their CFA or HRA within a single year was calculated. Subsequently, in order to evaluate overlap in space use between years, the number of years that each 5 × 5 km grid cell was used was calculated. Additionally, for each pairwise combination of 2 years, and in both directions, the proportion of cells that were used in the populations mean HRA and CFA in year X that were also used in year Y was calculated in order to investigate the sample overlap in foraging locations between specific years. Given that the sample of the population we tracked did not represent the entire population, we calculated the population overlap using the equation.

$${\text{Population overlap}} = O \times 100/S_\text{Y2}$$

where *O* is the sample overlap (%) and *S*_Y2_ is the percentage of the total predicted HRA or CFA in our second year sample (See Appendix 1). This calculation assumes that for both CFA and HRA areas which are visited but not observed are as likely to have been visited as those which have been visited and observed, i.e. detection rate is equal in overlapping, and non-overlapping cells.

Foraging habitats of northern gannets have previously been linked to chlorophyll a, sea surface temperature, bathymetry and copepod abundance (Hamer et al. 2000; Scott et al. 2013; Votier et al. 2010). Thus, further evidence to support these links is not addressed in this study. Additionally, this study deals with predicted population metrics, and thus, an index of oceanographic conditions at a larger scale is more relevant; therefore, the June NAO index, downloaded from www.cgd.ucar.edu/cas, was used as an index of annual oceanographic conditions. Warmer sea temperatures in years with a high NAO index influence the type and abundance of fish communities (O’Brien et al. 2000; Planque and Taylor 1998), which in turn influence the foraging behaviour of seabirds (Garthe et al. 2011).

## Results

**Fig. 4:**
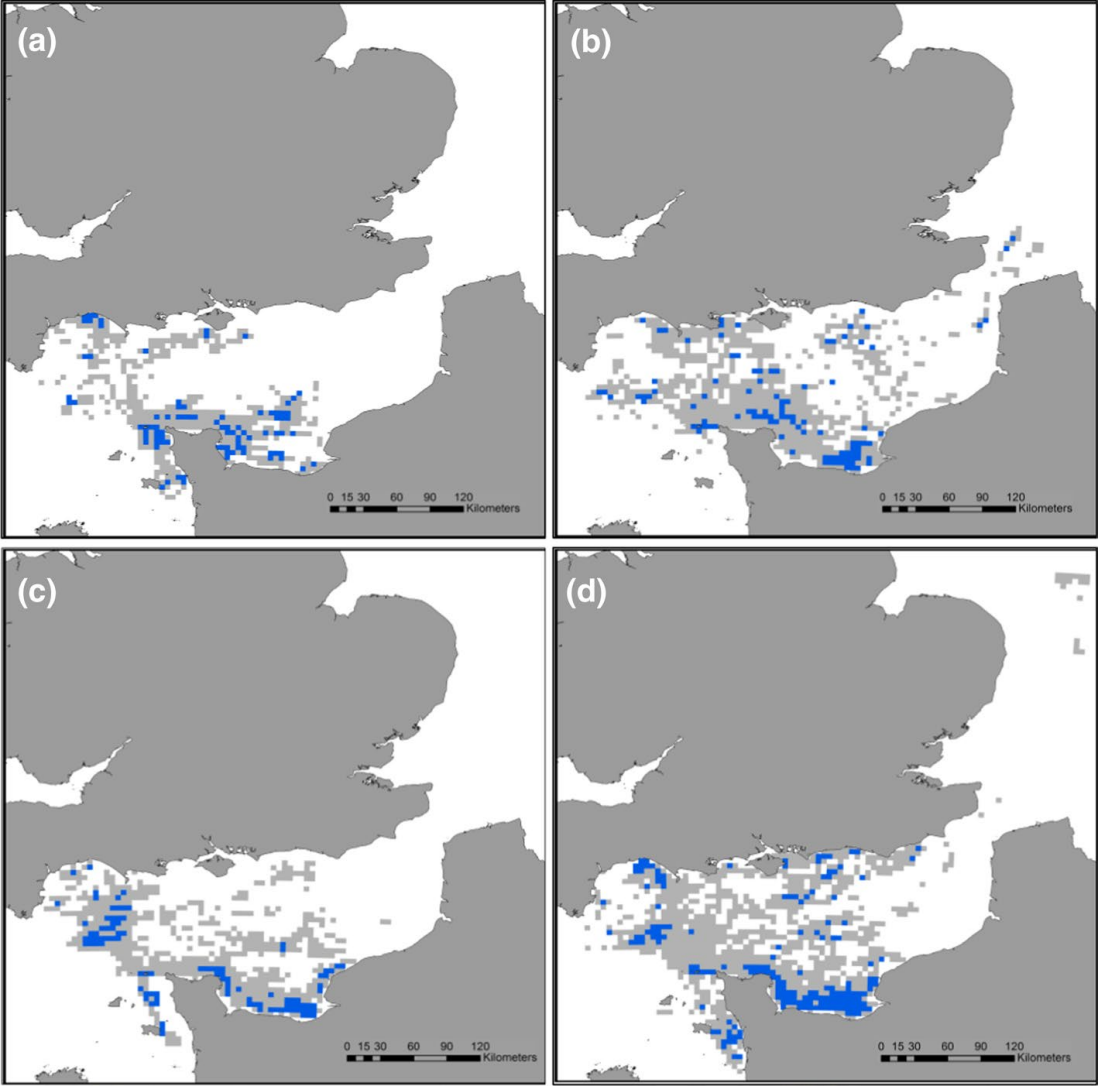
Proportion of time spent in the core foraging range (50 %—*blue cells*) and home range (95 %—*grey cells*) of a sample of northern gannets breeding on Alderney, Channel Islands, in **a** 2011, **b** 2013, **c** 2014, **d** 2015. This approach combines the data from all birds in order to calculate the time in area for each year

Northern gannets tracked on Alderney between 2011 and 2015 consistently foraged within the English Channel, though they were also recorded, on occasion, in the North Sea (Fig. 4). From 2011 to 2015, mean (±SE) trip duration changed from 16.6 ± 2.1 to 27 ± 1.4 h, corresponding to a shift in mean length from 331 ± 34 to 476 ± 22 km, and mean maximum distance to the colony from 106 ± 9.9 to 135 ± 7 km, respectively. Northern gannets overlapped with windfarm sites less often in 2011 and 2014, than in 2013 and 2015 (Table 1).

**Table 1:** Number and proportion of northern gannets breeding on Alderney, Channel Islands, and their foraging trips, which overlap with windfarms proposed for development in the English Channel

Year	Birds			Trips		
	*N*_windfarm_	*N*_total_	%	*N*_windfarm_	*N*_total_	%
2011	3	17	18	4	37	11
2013	9	15	60	24	72	33
2014	9	13	69	18	83	22
2015	13	15	87	33	96	34

### Inter-annual variation in foraging areas

**Fig. 5:**
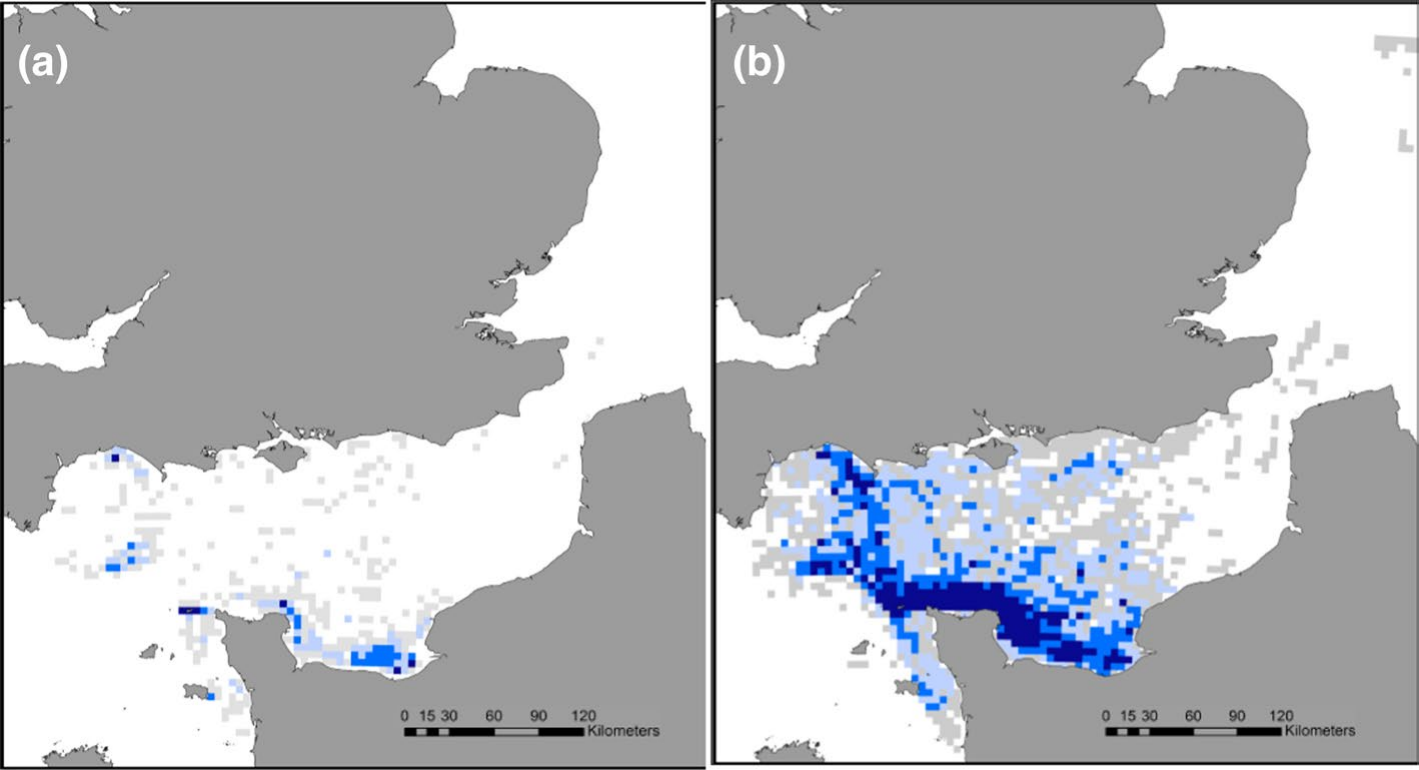
Overlap of **a** 50 % core foraging area and **b** 95 % home range cells used by the tracked sample of northern gannets breeding on Alderney in 1 (*grey*), 2 (*pale blue*), 3 (*mid blue)* or all 4 (*dark blue*) years of study

Both the CFA and the HRA of tracked gannets varied between years (Fig. 4). While commonly used areas around the North coast of France in the CFA and around Alderney in the HRA were observed in multiple years, sampled birds used relatively few areas consistently in all 4 years of study, especially in terms of CFA (Fig. 5). Scaling these samples up to population-level predictions also revealed differences between years in the extent of predicted CFA and HRA (Table 2). Predicted CFA was greater in 2015 than 2011, 2013 and 2014, respectively, with an increase in size of 30 % from smallest to largest. Similarly the predicted HRA was greater in 2015 than 2013, 2014 and 2011, respectively, with an increase in size of 60 % from smallest to largest (Table 2).

**Table 2:** A tracked sample of northern gannets and indices to measure how well the sample each year represents the 50 % home range (HRA) and the 95 % core foraging area (CFA) of the entire population (~10,000 birds)

Year		Number of birds	Number of trips recorded per bird	CFA *a*	HRA *a*	CFA *b*	HRA *b*	CFA Km^2^	HRA Km^2^	Prop. of total CFA sampled	Prop. of total HRA sampled	No. of individuals needed to represent 95 % of CFA	No. of individuals needed to represent 95 % of HRA	*P*	NAO June
2011	Sample	17	2.2	6987	21,871	35.7	14.4	2254	11,823	0.32	0.54	633	267	na	−1.15
	Population	10,000						6962	21,840						
2013	Sample	15	4.8	6175	30,010	14.8	8.5	3103	19,126	0.50	0.63	274	160	0.51	0.59
	Population	10,000						6166	29,989						
2014	Sample	13	6.4	5455	25,647	11.7	8.2	2874	15,683	0.53	0.61	217	155	0.61	−0.58
	Population	10,000						5449	25,627						
2015	Sample	15	6.4	7026	34,830	10.2	6.9	4287	24,296	0.61	0.70	191	130	0.48	0.17
	Population	10,000						7019	34,803						

*a* is the asymptote value (i.e. the predicted size of the CFA/HRA for the entire population), *b* is the value of *x* (i.e. the number of individuals) at which half of the maximum response is attained and both are derived from the Michaelis–Menton equation. *P* is reproductive success, and the NAO index for June each year is also shown

A similar pattern was seen in terms of population and sample overlap in the number of grid cells used in different years. For CFA, 2015 encompassed a greater proportion of cells than the other 3 years (Table 3). More dramatically, HRA in 2015 was predicted to encompass all of the cells also predicted to be used by the birds in 2014 and 2011, and nearly all of those used in 2013 (Table 3). A value of >100 % was calculated for the population overlap as a result of the slight discrepancies when extrapolating up from the sample overlap. The value of *b* from the Michaelis–Menten equation, which indicates how similar birds are to each other in their foraging areas, also varied between years (Table 2). Birds from 2015 were the most similar to each other (lowest value of *b*) for both CFA and HRA and in 2011 were the most different.

**Table 3:** Inter-annual population (and sample) overlap (%) in the 5 km by 5 km grid cells used in the CFA and HRA of a population of northern gannets breeding on Alderney, Channel Islands

	2011	2013	2014	2015
CFA				
2011	X	30 (15)	28 (15)	52 (32)
2013	41 (13)	X	51 (27)	66 (40)
2014	41 (13)	55 (28)	X	87 (53)
2015	47 (15)	46 (23)	55 (29)	X
HRA				
2011	X	90 (57)	89 (54)	102 (71)
2013	67 (36)	X	74 (45)	92 (64)
2014	78 (42)	87 (55)	X	107 (75)
2015	63 (34)	78 (49)	77 (47)	X

### Inter-annual variation in foraging trip characteristics

**Fig. 6:**
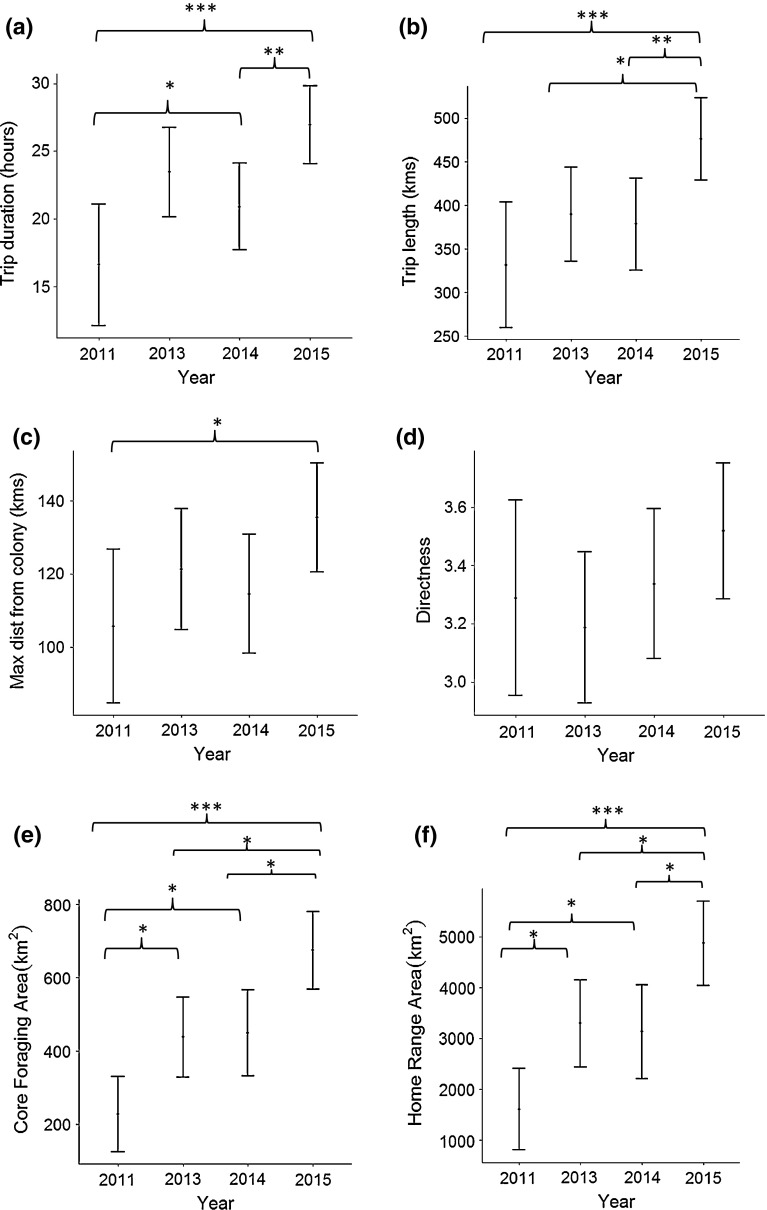
Inter-annual variation in the least squares mean (standard errors) values for **a** trip duration (T_283_ = 3.85, *p* < 0.001), **b** trip length (T_283_ = 3.83, *p* < 0.001), **c** maximum distance from the colony (T_283_ = 2.71, *p* < 0.01), **d** directness (T_283_ = 1.86, *p* = 0.06) **e** core foraging area (T_60_ = 5.7, *p* < 0.001), **f** home range area (T_60_ = 5.2, *p* < 0.001) from a sample of northern gannets breeding on Alderney, Channel Islands. Significant differences between years calculated from post hoc tests are displayed on the graph. The *brackets* indicate significant differences between the two end points of each bracket. The *asterisks* indicate levels of significance: *0.05, **0.01, ***0.001

We found strong evidence of inter-annual variation in trip duration, trip length, maximum distance from the colony, core foraging area and home range area from the tracked gannets (Fig. 6). In addition there was weak evidence of inter-annual variation in the directness of foraging trips (Fig. 6). Broadly speaking, trips in 2015 were longer in duration, distance travelled, maximum distance from the colony, directness, and birds had larger CFA and HRA than 2013, 2014 and 2011, respectively. Correspondingly, the June NAO index was negative in 2011 and 2014 and positive in 2013 and 2015, also coinciding with lower reproductive success in 2013 and 2015 (Table 2).

**Fig. 7:**
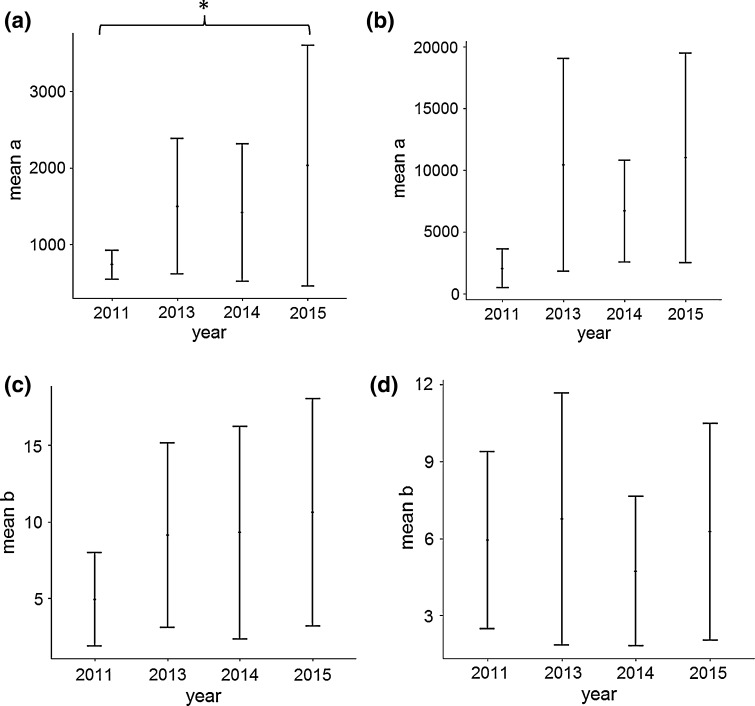
Mean size (*a*) of the **a** core foraging area (T_42_ = 2.17, *p* = 0.036) and **b** home range area (T_42_ = 1.63, *p* = 0.11) and the mean number of trips per individual (*b*) necessary to reach half of the **c** core foraging area (T_42_ = 0.92, *p* = 0.37) and **d** home range area (T_42_ = 0.12, *p* = 0.90) for an individual bird, predicted using the Michaelis–Menton equation from a sample of tracked northern gannets from Alderney, Channel islands

The sample CFAs and HRAs of individual tracked birds overlapped with each other more often in 2015 than in 2011, with 2013 and 2014 having intermediate amounts of inter-individual overlap (Table 4). Additionally, there was inter-annual variation in the size of the predicted CFA for individual birds (Fig. 7a), and the higher mean and larger error bars in 2015 suggest that the CFA for individual birds was larger with higher inter-individual variation in size than in subsequent years. The predicted HRA for individual birds was not significantly different between the years; however, the large variation within years in these values suggests that the inter-individual variation in the size of HRA was also considerably higher in 2013 and 2015 (Fig. 7b).

**Table 4:** The Overlap between individuals in the number of 5 km by 5 km grid cells used in the core foraging area and home range area for northern gannets tracked from Alderney, Channel Islands in a single year

Year	Number of cells used by n birds															
	1	2	3	4	5	6	7	8	9	10	11	12	13	14	15	16
CFA																
2011	124	14	1	0	0	0	0	0	0	0	0	0	0	0	0	0
2013	195	25	4	0	0	1	0	0	0	0	0	0	0	0	0	0
2014	175	18	5	2	0	0	0	0	0	0	0	0	0	0	0	0
2015	273	45	16	4	1	0	0	0	0	0	0	0	0	0	0	0
HRA																
2011	391	134	52	23	7	10	5	4	0	1	0	0	0	0	0	1
2013	575	293	89	60	24	11	6	2	2	0	2	1	0	1	0	0
2014	490	193	81	43	26	11	7	3	5	1	0	0	1	0	0	0
2015	694	358	185	83	30	27	21	11	13	8	4	0	0	0	1	1

The number of trips necessary for a sample to represent half of both CFA and HRA for individual birds (*b*) predicted using the Michaelis–Menten equation did not vary significantly between the years, suggesting that between trips individual birds were similarly consistent in their habitat use between years. However, the within-year variation surrounding these values represents the inter-individual variation in consistency, i.e. some birds were very consistent in their foraging locations, whereas others were more variable. This variation was also lowest in 2011 and 2014 which suggests there was smaller inter-individual variation in the consistency of the location of HRA of individuals in those years (Fig. 7d).

## Discussion

Seabirds are known to exhibit inter-annual variation in foraging behaviour at the population level, and intra- and inter-individual flexibility; however, few studies link the two. We show strong evidence of inter-annual variation in the size and location of core foraging areas and in foraging trip characteristics recorded from a sample of northern gannets breeding on Alderney, Channel Islands. Gannets tracked in 2015 undertook trips with a longer duration, length and maximum distance from the colony as well as larger CFA and HRA than those recorded in other years. This corresponded with a lower breeding success than previously recorded. This large foraging range in 2015 combined with the largest overlap of HRA and CFA between individuals suggests that all individuals travelled extensively in search of prey. Thus, inter-annual variation in the size of the foraging area for the entire population is driven by individual birds visiting larger areas in all of their trips, not by individual birds diversifying to visit more, different areas (Fig. 1), which is indicative of low prey availability.

### Inter-annual variation in foraging areas and trip characteristics

Variation in physical oceanographic processes can alter the distributions of plankton and fish and, thus, prey availability to seabirds (Shealer et al. 2002) resulting in inter-annual variation in foraging locations for many species (Burke and Montevecchi 2009). Seabirds have developed a flexible foraging strategy as a mechanism with which to deal with this spatial and temporal variation in prey distribution (Montevecchi et al. 2009; Weimerskirch et al. 2005) and the inter-annual variation in foraging areas and trip characteristics of Alderney’s northern gannets may be explained by this.

Reduced prey availability can result in longer foraging trip duration, range and core foraging area in seabirds (Monaghan et al. 1994; Suryan et al. 2000). Thus, the longer foraging trips and larger CFAs from gannets tracked in 2015 than those tracked in 2011 and 2014 may be due to lower prey availability as a result of oceanographic conditions (Burke and Montevecchi 2009). The June NAO index in 2013 and 2015 was higher than in 2011 and 2014 (Table 2), which is consistent with years of increased trip duration and range. This suggests that the NAO might be influencing the type, abundance and availability of prey and, thus, seabird foraging behaviour in the English Channel. Sea temperature may influence the structure of fish communities (Perry et al. 2005), and in warmer temperatures some prey may occur deeper, potentially becoming unavailable to seabirds (Montevecchi 2012). Links between NAO and the distribution of other seabirds such as Cory’s Shearwater *Calonectris borealis* (Paiva et al. 2013a) and Macaronesian Shearwater *Puffinus baroli* (Ramos et al. 2015) have been shown. Additionally, northern gannets have been observed to travel further with a larger home range in years where larger pelagic fish were more abundant than small fish (Garthe et al. 2011), potentially explaining the larger CFA and HRA in 2015 when the NAO index was high. However, the NAO index was even higher in 2013, and although trip duration was longer and CFA and HRA were larger than in 2011 and 2014 when the NAO indexes were negative, they were not as extreme as in 2015; this suggests that other factors, such as increased fishing activity, or increased patchiness of prey, were also involved; however, we could not evaluate this further within the scope of our study.

The combination of the increased foraging range and large overlap of HRA and CFA between individuals in 2015 implies that all individuals had large searching areas, i.e. all birds were going everywhere in search of prey (e.g. Fig. 1b), rather than to consistent individual-specific foraging areas (e.g. Fig. 1d). This suggests that prey was widespread and thinly dispersed, which is consistent with the less direct path between the colony and foraging areas observed in that year. Gannets showed the most direct path between the colony and the foraging areas in 2013, again suggesting that prey may have been in more predictable locations in that year (Pettex et al. 2010). Trip duration was higher and CFA and HRA smaller in 2013, than in 2011 and 2014 and this, combined with a more direct commuting path, suggests that gannets were foraging in more predictable locations, further from the colony in 2013 (e.g. Fig. 1a). However, the directness of foraging trips may also be related to other behaviours such as wind direction (Gremillet et al. 2004), or following fishing vessels (Votier et al. 2010), or conspecifics (Buckley 1997). The lower HRA combined with fairly direct trips and shorter trip durations in 2011 and 2014 suggest that birds were foraging at predictable locations with higher prey availability closer to the colony in these years.

Breeding success was also lower in 2013 and 2015 than in 2014 and may be a result of the increased foraging trip duration in those years. If adults have had to travel further from the colony in order to forage, they may have failed to return with sufficient food for chick provisioning (Baird 1990), or at a sufficient rate in order to maximise reproductive success (Suryan et al. 2002). Additionally chicks left unattended at the colony are open to attacks by predators or conspecifics (Lewis et al. 2004).

In general, there was little overlap in the locations of sampled CFA between years, with only 8 of the 5 km by 5 km cells being used in all 4 years. This suggests that the distribution of prey varied between the years. However, the 5 km × 5 km cells used for these analyses are small in comparison with the scale of some Area Restricted Search (ARS) behaviour observed in gannets (Hamer et al. 2009); thus, overlap in foraging location at these larger scales is omitted. However, a previous study of the foraging behaviour of Alderney’s gannets found that this was the most efficient scale to capture their search behaviour (Warwick-Evans et al. 2015). Furthermore, we know that our sample under-represents the population CFA and HRA and that sample overlap is thus lower than population overlap (Table 2). Thus, we can assume that more cells are actually visited in multiple years. Overlap in HRA between the years was much larger, as birds tended to commute along similar paths to reach foraging areas, particularly towards Northern France and south-west UK where foraging occurred in all 4 years. In fact, sampled birds in 2015 used all of the HRA cells used in 2011 and 2014, and most of those used in 2013. This is further evidence that it was necessary for these gannets to travel further in order to forage in 2015, and thus, prey items were more widely dispersed.

### Intra- and inter-individual variation

Gannets tracked on Alderney in 2011 required fewer trips to be tracked in order to represent half of the CFA of individual birds than in subsequent years, i.e. these birds displayed lower intra-individual variation (higher consistency) in the location of the CFA of individual trips than those tracked in later years (Fig. 7c). However, these results were not significant, probably due to the low sample size of individuals with multiple trips recorded in this year. The values of *b*, in terms of CFA, were similar amongst the subsequent 3 years, and thus, inter-annual variation in this intra-individual variation cannot be confirmed. The low inter-annual variation in *b* in terms of HRA illustrates that intra-individual variation in the location of the HRA was similar between years. However, the variability in this value, described by the error bars, was considerably larger in 2013 and 2015, than 2011 and 2014, demonstrating higher inter-individual variation in their intra-individual variation in 2013 and 2015.

Gannets tracked on Alderney in 2015 displayed lower inter-individual variation in the locations of CFAs and HRAs than in previous years, as described by the low b value (Table 2). Low levels of inter-individual variation in the location of CFAs suggest either that prey is concentrated in small areas, attracting all individuals (e.g. Fig. 1a), or that prey is sparsely distributed and all individuals in the population have large searching areas. The low inter-individual variation observed in 2015 combined with the larger CFA strongly suggests that, of these two alternatives, this inter-individual variation was driven by individual birds visiting larger areas (Fig. 1b). Combining this low inter-individual variation with the large overlap in CFA between sampled individuals in that year, we can suggest that the inter-annual variation in the size of the CFA for the entire population is also driven by individual birds visiting larger areas, and not by individual birds visiting more, different areas (Fig. 1d).

Consistency in foraging locations within and between individuals has been shown in northern gannets (Patrick et al. 2014; Wakefield et al. 2015) and other seabirds (Irons 1998; Weimerskirch 2007) and may be due to individual specialisation in diet (Bearhop et al. 2006; Patrick et al. 2014; Woo et al. 2008) or predictability of prey patches (Hamer et al. 2001; Weimerskirch 2007). However, this consistency is rarely considered at an inter-annual level, although Wakefield et al. (2015) demonstrated that gannets show intra-individual consistency in foraging areas across years, due to long term dietary specialisation, and site familiarity gained in early life. Our data suggest that in the more challenging foraging conditions of 2013 and 2015, more individuals in the population were generalist in terms of foraging locations, however this may be due to selecting different proportions of individuals with different foraging strategies, in terms of generalist or specialist, in different years.

### Limitations and implications

Predictions from the Michaelis–Menten equation indicate that in no year did our sample of gannets fully represent either the HRA or CFA predicted for the entire population breeding on Alderney. This is likely to be the case in the majority of seabird tracking studies as devices can be costly, and logistics of getting to colonies may limit the frequency of fieldwork, which can result in only sampling a small proportion of the population. The relative importance of this limitation depends on the question being asked. If differences in the trip characteristics between groups, for example males and females (e.g. Cleasby et al. 2015), are being investigated, then it could be assumed that under-representation of the entire population in terms of trip characteristics would not be biased in either direction, and thus would not influence the conclusions. However, if the location of CFAs or HRAs is being explored, then this can have important consequences, particularly if tracking studies are being used to identify important areas for conservation or marine spatial planning. For example, the proportion of birds/trips entering windfarm sites in this study are also likely to be underestimated, which, in turn, may have implications when predicting the impacts from these devices.

In this study, the number of birds necessary to track in order to represent the CFA for the entire population varied annually, as a result of differences, between years, in the inter-individual variation in the location of CFA. It would have been necessary to track many more birds in 2011 than in the subsequent years. However, only 2.4 trips per individual were recorded in 2011, considerably fewer than in subsequent years, and this supports the idea that gannets display intra-individual variation in foraging locations and highlights the importance of sampling multiple trips per individual (Soanes et al. 2013a). This inter-annual variation in the number of birds necessary to track to represent the CFA of the whole population was also observed in years where similar numbers of trips per individual were recorded (2013–2015). This indicates that inter-individual variation in the location of CFA differs between years, and should be an important consideration in tracking studies.

Gannets tracked in 2015 undertook foraging trips with a longer duration and length and a larger CFA and HRA than gannets tracked in previous years. These inter-annual differences in foraging behaviour are driven by differences in the intra- and inter-individual variation in foraging behaviour and location between the years, and may be associated with variation in oceanographic conditions, and a lower breeding success (Becker et al. 2007; Garthe et al. 2011). Years with sparsely distributed or low abundance of prey, may become more frequent as a result of exploitation by commercial fisheries or climate change (Perry et al. 2005). This may result in increased trip duration, potentially leading to lower reproductive success through both energy limitation and predation or competition (Lewis et al. 2004). Additionally, if core foraging areas and home range areas of individual birds increase, then more individuals are likely to encounter pressures from spatially variable anthropogenic disturbances, such as the development of windfarms. Indeed, gannets tracked in this study overlapped with windfarm sites less often in 2011 and 2014, than in 2013 and 2015, in terms of both birds and trips.

Furthermore, intra-specific competition from the large North Sea gannetries may limit the foraging range of Alderney’s gannets (Wakefield et al. 2013). Interestingly, Alderneys gannets show consistency in their westward boundaries, most likely because the gannets from Les Sept Iles forage in the western English Channel (Gremillet et al. 2006), thus limiting the potential range of Alderney’s gannets. If North Sea gannets limit the northern boundaries, then Alderney’s gannets may be forced to alter their time budgets or prey type in years of poor food availability. This may have negative impacts on reproductive success, as alternative prey items may have a lower energetic value, or altered time budgets may be more energetically costly.

## Appendix: Overlap equation

The equation to calculate the overlap in cells used for the CFA/HRA between years, for the entire population is: Population overlap = O × 100/S_Y2_, where O is the observed overlap (i.e. the % overlap in cells used for the CFA/HRA between years in our tracked birds) and S_Y2_ is the percentage of the total predicted CFA/HRA sampled in year 2. Y1 and Y2 represent year 1 and year 2 respectively. This equation was derived from a series of possible overlap scenarios: 1: If a cell was used in the CFA/HRA in year 1 and year 2 (Population overlap = Y1Y2) then the cell could be observed in year 1 and year 2 (Y1Y2), observed only in year 2 (– Y2), observed only in year 1 (Y1 – ), or not observed at all (– –). Alternatively, if a cell was used in the CFA/HRA in year 1 but not in year 2 (population overlap = Y1 –) then the cell could be observed only in year 1 (Y1 – ), or not observed at all (– –).

Let *q* be the probability that a cell that was visited in Y1 was also visited in Y2 (i.e. the population overlap), let *p*_*1*_ be the probability of observing a cell that was visited in year 1, and *p*_*2*_ be the probability of observing a cell that was visited in year 2 (i.e. the proportion of the total predicted HRA or CFA that was sampled). Then the probability of each outcome can be calculated as follows:

Thus, the proportion of cells observed in year 2 which overlap with cells observed in year 1 = *n*_*1*_/(*n*_*1*_ + *n*_*3*_) (i.e. the number of cells visited and observed divided by the total number of cells visited, whether or not they were observed), thus the percentage overlap (O) = 100 × *n*_*1*_/(*n*_*1*_ + *n*_*3*_). We know that *n*_*1*_ = *nqp*_*1*_*p*_*2*_, where *n* is the total number of cells and that

$$ \begin{aligned} n_{3} = & n(qp_{1} \left( {1 - p_{2} } \right) \, + \, \left( {1 - q} \right)p_{1} ) \\ = & n\left( {qp_{1} - \, qp_{1} p_{2} + \, P_{1} - \, qp_{1} } \right) \\ = & n\left( {p_{1} - \, qp_{1} p_{2} } \right) \\ = & np_{1} (1 - qp_{2} ) \\ \end{aligned} $$

Thus

$$ \begin{aligned} {\text{O}} = & 100 \times nqp_{1} p_{2} /(n(qp_{1} p_{2} + \, p_{1} (1 - qp_{2} ))) \\ = & 100 \times nqp_{1} p_{2} /\left( {np_{1} } \right) \\ = & 100 \times qp_{2} , \\ \end{aligned} $$

so *q* = O/(100 × *p*_*2*_). We know that S_Y2_ = *p*_*2*_ × 100

Thus population overlap (%) = O × 100/S_Y2_.
